# Reporting of pain by people with chronic obstructive pulmonary disease (COPD): comparative results from the HUNT3 population-based survey

**DOI:** 10.1186/s12889-018-5094-5

**Published:** 2018-01-25

**Authors:** Randi Andenæs, Astrid Momyr, Idunn Brekke

**Affiliations:** Faculty of Health Science, Department of Nursing and Health Promotion, Oslo Metropolitan University, PB 4 St.Olavs plass, N-0130 Oslo, Norway

**Keywords:** Epidemiology, Population comparison, Arthritis, Heart disease, Diabetes

## Abstract

**Background:**

Chronic obstructive pulmonary disease (COPD) is often associated with chronic pain, but pain in COPD remains poorly understood, particularly in comparison to pain in other groups. We compared the pain reported by people with COPD with that reported by arthritis, heart disease, diabetes, and those not reporting any disease, while adjusting for the effects of selected sociodemographic and lifestyle factors, comorbidities, anxiety, and depression.

**Methods:**

Using cross-sectional data from a population-based health survey in Norway (HUNT3; *n* = 50,807), we included participants with COPD (*n* = 1199), participants without COPD, but with arthritis (*n* = 8582), heart disease (*n* = 4109), or diabetes (*n* = 1254), and participants without any disease (*n* = 18,811). Logistic and linear regression analyses were performed to estimate the probability of reporting chronic pain and the level of pain intensity in the different groups adjusting for other relevant factors.

**Results:**

Approximately half (51.8%) of people with COPD reported chronic pain, which was a significantly higher rate than in the diabetes and non-disease groups, and similar to the heart disease group. People with arthritis had a chronic pain rate of 75.4%, which was higher than all other groups, including COPD. Analyses of pain intensity yielded similar findings, with the COPD group having higher pain intensity than the diabetes and non-disease groups, similar pain intensity as the heart disease group, and less pain intensity than the arthritis group. The likelihood of chronic pain and the intensity of pain were generally higher among women, people employed in occupations with low educational requirements, smokers, and those with comorbidity. Chronic pain rates and pain intensity increased with age and higher anxiety and depression scores, and were inversely related to physical activity.

**Conclusions:**

People with COPD are at increased risk for chronic pain and higher pain intensity, second only to those with arthritis among the disease groups included in this study. The findings indicate a close relationship between pain and anxiety and depression. The relationships between pain and socioeconomic and lifestyle factors (e.g., smoking and exercise) suggest the need for efforts at the societal level to reduce inequality in health.

## Background

Chronic obstructive pulmonary disease (COPD) is a lung disease characterized by persistent airflow obstruction and chronic inflammation of the airways [1]. COPD is among the four major non-communicable diseases, along with cardiovascular disease, cancer and diabetes, that are responsible for 82% of deaths from non-communicable disease [2]. Although COPD mortality rates have declined in recent years due to improvements in COPD management [3], the condition remains a major burden for both the individual and the healthcare system, as well as a significant contributor to societal healthcare costs. In addition, comorbidities commonly associated with COPD, such as anxiety and depression, can exacerbate COPD severity and its impact on both the individual and society [4]. Shortness of breath is the key symptom of COPD [1], but pain has gained increasing attention in recent years and is now recognized as a significant problem for people with COPD [5, 6]. The reported prevalence rates of pain in people with COPD are highly variable, ranging from 32% [7] to 82% [8]. Pain severity is reported to be in the moderate to severe range [9–13]. Common locations for pain are in the neck, shoulder blades and lower back [8]. Roberts et al. [14] showed that pain among COPD patients is most commonly inflammatory pain, with mechanical/compressive back pain being the second most common [14]. The systemic inflammatory process in COPD, which activates cytokines, may be the underlying cause of chronic and neuropathic pain [6].

Most published studies of pain in people with COPD have been restricted to clinical or pulmonary rehabilitation settings and have included small sample sizes. By contrast, Roberts et al. [14] studied a large cohort of 7952 patients with COPD and a matched control group of 15,904 patients with other chronic diseases (non-COPD). Comparison of the COPD and non-COPD groups showed a significantly higher percentage of patients with chronic pain in the COPD group (59.8% vs. 51.7%, respectively) [14].

In primary care, chronic pain is a commonly reported symptom and represents a significant public health concern [15]. The prevalence of chronic pain is high, although European population estimates vary widely—from 2 to 51% [16, 17]. The 1-month prevalence of moderate-to-severe non-cancer-associated chronic pain has been reported to be 19% in Europe [18]. It has been suggested that, compared with other health problems, pain has the greatest impact on quality of life [19]. Chronic pain places a substantial burden on people, influences their overall perceptions of general health, is associated with depressive symptoms, and negatively affects their work, daily activities and social relationships [18].

For people with medical conditions such as arthritis, various heart diseases, or diabetes, chronic pain is a common symptom [20], although prevalence estimates differ greatly across groups [21]. Few studies have compared the prevalence of chronic pain in people with COPD and those with either the aforementioned diseases, representing the most widespread chronic diseases, or those without any medical conditions. Better understanding of the epidemiology of pain, both within and across disease and non-disease groups, is necessary for developing health care services that are more closely attuned to patients’ specific needs. Thus, the objective of this study was to investigate whether people with COPD are at higher risk for chronic pain and report higher pain intensity compared with those with arthritis, heart disease, diabetes, or no disease, adjusting for selected confounding variables, including demographic and lifestyle variables, comorbidities, anxiety, and depression.

## Methods

### Data source

In this study, we analyzed cross-sectional data obtained from the HUNT Study in Norway, which with 50,807 participants, is one of the largest health studies ever performed. The third health survey (HUNT3) was carried out in 2006 to 2008 and invited all inhabitants of the county of Nord-Trøndelag aged 20 years and older to participate. The health study included questions about socioeconomic conditions, health-related behaviors, symptoms, illnesses, and diseases. The general design and details of the HUNT3 study are given elsewhere [22, 23]. The county of Nord-Trøndelag is representative of Norway in terms of geography, economy, industry, sources of income, age distribution, and the morbidity and mortality rates of its inhabitants [24, 25].

### Participants and sample

Of the 90,860 people invited, 50,807 participated, yielding a 54.1% participation rate. For the purpose of the present study, we included participants with a non-communicable chronic diseases (COPD, arthritis, heart disease, or diabetes), and healthy people who reported no disease (*n* = 18,811). In the questionnaire, medical conditions were assessed by self-report, with respondents indicating whether they have or ever had any of the 20 listed medical conditions. Cancer was not included as a specific disease group in this study because, due to the wording of the disease questions, respondents with a current diagnosis of cancer could not be distinguished from those who were successfully treated in the past and did not currently have chronic disease.

Because respondents could indicate more than one disease, we grouped them into mutually exclusive categories using a new variable named “disease”. The variable “disease” was coded as follows: All respondents who indicated chronic bronchitis, emphysema, or COPD were defined as having COPD (*n* = 1199) and were coded as 0. Those who indicated Rheumatoid arthritis, osteoarthritis, Bechterew’s disease, or fibromyalgia, but not COPD, were defined as having arthritis (*n* = 8582) and were coded as 1. Those who indicated myocardial infarction, angina pectoris, cardiac insufficiency, or unspecific heart disease, but not COPD or arthritis, were defined as having heart disease (*n* = 4109) and were coded as 2. Those who indicated diabetes, but not COPD, arthritis, or heart disease, were defined as having diabetes (*n* = 1254) and were coded as 3. Those who did not indicate any of the medical diseases listed in the questionnaire composed the non-disease group (*n* = 18,811) and were coded as 4. These five groups, totaling 33,955 respondents, constitute the sample in the present study.

### Measures

#### Pain

To assess for chronic pain, participants answered “yes” or “no” to the question “Do you currently have bodily pain now that has lasted more than 6 months?” This definition is stricter than that of the International Association of the Study of Pain, which defines chronic pain as “pain that persists beyond normal tissue healing time, which is assumed to be 3 months” [26]. Participants were also asked to rate the intensity of bodily pain during the past 4 weeks as no pain (0), very mild (1), mild (2), moderate (3), severe (4), or very severe (5).

#### Anxiety and depression

The Hospital Anxiety and Depression Scale (HADS) [27] is a two-dimensional self-report instrument for assessing anxiety and depression. It includes 14 items that measure symptoms during the past week: seven for the anxiety subscale and seven for the depression subscale. Each question is scored as 0–3, with a maximum score of 21 for each subscale; a higher score indicates greater anxiety or depression. Previous studies and reviews have concluded that the HADS is a reliable and valid instrument for assessing the separate dimensions and symptom severity of anxiety and depression in somatic, psychiatric, and primary care patients, as well as in the general population [28].

#### Comorbidity

Comorbidity was based on the list of 20 medical conditions and was divided into two categories: 1) *no comorbidity*, defined as having no disease other than the one specified in the “disease” variable, and 2) *comorbidity*, defined as having one or more of the 20 diseases listed in the questionnaire *in addition to* the one specified in the “disease” variable. Because the non-disease group was defined as having none of the 20 listed diseases, none of the participants in the non-disease group were categorized as having comorbidity.

#### Sociodemographic and lifestyle variables

Information on age, sex, and living arrangement (living alone or with a partner) was recorded for each participant. Since the HUNT survey does not have any information on educational level, occupation was chosen as a proxy. Occupation was recoded according to the original 10-category work title classification used by Statistic Norway [29] and divided into two categories: 1) profession or employment with a high educational requirement (e.g., leaders, academics, politicians), and 2) profession or employment with a low educational requirement (e.g., industry workers, care takers, sales and service). The question about smoking status asked participants to indicate whether they were a previous smoker, smoke daily, smoke occasionally, or never smoke. Physical activity was assessed in a question about the respondent’s average number of hours of physical activity per week (never, less than once a week, once a week, 2–3 times a week, or nearly every day). Examples of activities were walking, skiing, swimming, or participation in exercise/sports.

### Statistical analyses

Descriptive summary of the variables included in the variables were presented as percentages, mean with standard deviation (SD). Bivariate analysis of disease group and pain was performed with cross-tabs using chi-square test. The association between chronic pain and independent variables was analyzed using logistic regression, with odds ratios (ORs) and 95% confidence intervals (CIs). Chronic pain was treated as a categorical variable coded as 1 if the person reported pain lasting more than 6 months and 0 otherwise. The second dependent variable, pain intensity, was analyzed using ordinary linear regression, with regression coefficients, standard errors, and *p*-values. Pain intensity was rated on a six-point pain intensity scale with 0 representing no pain and 5 representing very severe pain. The models for both dependent variables (chronic pain and pain intensity) included the same independent variables. There were some missing values in the dataset. Excluding missing cases or including the missing cases as a separate category could have led to biased estimates. Therefore, multiple imputation was used to generate 5 imputed datasets using the mi impute chained command in Stata® 13. The procedure replaced each missing value with a set of plausible values based on all other variables included in the statistical model. We also ran the models without multiple imputation. The results of the analyses without imputed data (available upon request) were similar to those presented in Tables 2 and 3, in which we used multiple imputation. For further details, see White et al. [30].

## Results

### Descriptive summary

In this study, 3.5% (*n* = 1199; 51.7% women and 48.3% men) of the sample reported having COPD. The prevalence rates were 25.3% for arthritis (*n* = 8582; 70.4% women and 29.6% men), 12.1% for heart disease (*n* = 4109; 36.8% women and 63.2% men), and 3.7% for diabetes (*n* = 1254; 48.6% women and 51.4% men). All participants with heart disease and with diabetes reported comorbidity, while the COPD and arthritis group had 80.2 and 88.9, respectively. Table 1 shows that people with heart disease were the oldest on average (mean 68.9 years) and those with no disease were the youngest on average (mean 51.6 years). Raw scores (i.e., without controlling for age) indicated that people with heart disease were more likely than those in other groups to be employed in occupations with low educational requirements. The percentage of smokers varied widely. People with COPD had the highest prevalence (75.9%) of smoking either daily or occasionally, and the non-disease group had the lowest smoking prevalence (45.6%). Of the people with COPD, 10.7% reported that they never exercised. Anxiety and depression scores were highest in those with COPD.

**Table 1:** Sample characteristics of each disease group

	COPD	Arthritis	Heart disease	Diabetes	No disease
N (%)	*n* = 1199 (3.5)	*n* = 8582 (25.3)	*n* = 4109 (12.1)	*n* = 1254 (3.7)	*n* = 18,811 (55.4)
*Age, mean years (SD)*	61.6 (13.3)	61.2 (12.4)	68.9 (12.0)	63.4 (12.4)	51.6 (12.7)
*Female sex (%)*	51.7	70.4	36.8	48.6	48.8
*Housing*					
*Live with others (%)*	75.3	79.3	76.0	77.4	88.3
Missing observations, n	211	1356	592	185	3624
*Occupation*					
*With low educational requirements (%)*	78.9	77.1	81.0	78.5	72.3
*Smoking habits*					
*Previous smoker (%)*	18.6	37.4	32.4	41.4	47.2
*Smoke daily (%)*	44.8	31.5	47.7	38.8	29.7
*Smoke occasionally (%)*	31.1	20.4	14.1	14.6	15.9
*Never smoke (%)*	5.5	5.7	5.7	5.2	7.2
Missing observations, n	30	491	166	41	354
*Exercise*					
*Never (%)*	10.7	5.8	10.0	7.3	4.1
*Less than once a week (%)*	20.4	15.6	16.2	16.7	16.8
*Once a week (%)*	22.3	19.8	19.1	19.3	22.9
*2–3 times a week (%)*	32.3	38.1	33.8	36.5	39.7
*Nearly every day (%)*	14.2	20.7	20.9	20.2	16.5
Missing observations, n	0	404	136	29	135
*Comorbidities (%)*	80.2	88.9	100	100	–
*Hospital Anxiety and Depression Scale (HADS)*					
*HADS anxiety score, mean (SD)*	4.9 (13.8)	4.6 (3.6)	3.9 (3.3)	3.4 (3.1)	3.3 (2.8)
Missing observations, n	266	1765	833	258	4089
*HADS depression score, mean (SD)*	4.4 (3.2)	3.9 (3.1)	4.3 (3.1)	3.7 (2.9)	2.8 (2.6)
Missing observations	264	1687	762	242	4009
*Chronic pain (%)*	51.8	75.4	50.0	43.0	25.9
Missing observations, n	60	600	218	62	435
*Pain intensity*					
*No pain (%)*	19.5	8.3	23.5	32.1	42.0
*Very mild (%)*	9.7	6.1	9.8	10.2	13.2
*Mild (%)*	13.9	13.5	13.2	12.7	15.2
*Moderate (%)*	39.3	50.6	38.5	33.7	24.2
*Severe (%)*	15.8	19.4	13.3	10.1	4.8
*Very severe (%)*	1.8	1.9	1.7	1.2	0.5
Missing observations, n	91	718	375	140	1403

### Chronic pain in different disease groups

Participants with COPD were less likely to have chronic pain than those with arthritis, but more likely to have chronic pain than those with diabetes or no disease.

As shown in Table 1, the prevalence of chronic pain in the four disease groups ranged from a low of 43.0% in the diabetes group to a high of 75.4% in the arthritis group, with the non-disease group having a chronic pain prevalence of 25.9%. Chi-square test showed significant differences in chronic pain between the groups in the analyses (*p* < 0.001).

Table 2 shows the results of the adjusted logistic regression analysis of chronic pain in different disease groups, adjusted for confounding variables (age, sex, occupational status, living arrangement, lifestyle behavior, and anxiety and depression). To control for non-linear relationships, we also included the square of age in the model. Compared to the reference group of those with COPD, the odds of reporting chronic pain were much higher for those with arthritis (OR 2.81, 95% CI: 2.47–3.20) and lower for those with diabetes (OR 0.74, 95% CI: 0.63–0.88) and individuals without any disease (OR 0.64, 95% CI: 0.52–0.77). However, the odds of reporting of chronic pain did not differ significantly between those with COPD and those with heart disease.

**Table 2:** Adjusted logistic regression of chronic pain in different disease groups (*n* = 33,955)

*Independent Variables*	OR	CI 95%		*p*-value
*Disease group*				
*Arthritis*	2.81	2.47	3.20	≤ 0.001
*Heart diseases*	1.00	0.87	1.15	0.945
*Diabetes*	0.74	0.63	0.88	0.001
*No disease*	0.64	0.52	0.77	≤ 0.001
*COPD*	reference			
*Sex*				
*Men*	0.81	0.77	0.86	< 0.001
*Female*	reference			
*Age, years*	1.07	1.05	1.09	< 0.001
*Age squared*	0.99	0.99	0.99	< 0.001
*Housing*				
*Living with others*	1.05	0.97	1.13	0.196
*Living alone*	reference			
*Occupation*				
*With low educational requirements*	1.17	1.10	1.24	< 0.001
*With high educational requirements*	reference			
*Smoking habits*				
*Smoke daily*	1.30	1.22	1.37	< 0.001
*Previous smoker*	1.38	1.28	1.49	< 0.001
*Smoke occasionally*	1.18	1.06	1.32	< 0.001
*Never smoke*	reference			
*Exercise*				
*Less than once a week*	0.74	0.66	0.83	< 0.001
*Once a week*	0.64	0.57	0.72	< 0.001
*2–3 times a week*	0.63	0.56	0.71	< 0.001
*Nearly every day*	0.63	0.56	0.72	< 0.001
*Never*	reference			
*Comorbidity*	1.75	1.48	2.07	< 0.001
*Hospital Anxiety and Depression Scale (HADS)*				
*HADS anxiety score*	1.08	1.06	1.09	< 0.001
*HADS depression score*	1.05	1.04	1.06	< 0.001

Significance is set at *p* ≤ 0.05

Chronic pain was also associated with several other independent variables (Table 2). Men had lower odds of reporting chronic pain (OR 0.81, 95% CI: 0.77–0.86) than women. The odds of reporting chronic pain increased with increasing age (OR 1.07, 95% CI: 1.05–1.09) and was higher for those employed in occupations with low educational requirements (OR 1.17, 95% CI: 1.10–1.24) than for those employed in occupations with high educational requirements. Daily smoking was associated with much higher odds of reporting chronic pain (OR 1.30, 95% CI: 1.22–1.37) compared with people who did not smoke. Physical activity was negatively associated with the odds of reporting chronic pain. The odds of reporting chronic pain was lower in people who exercised nearly every day (OR 0.63, 95% CI: 0.56–0.72) than in those who never exercised. Those with comorbidity had higher odds of reporting chronic pain than those without comorbidity (OR 1.75, 95% CI: 1.48–2.07). The odds of reporting chronic pain also increased with increasing anxiety (OR 1.08, 95% CI: 1.06–1.09) and depression scores (OR 1.05, 95% CI: 1.04–1.06). Living arrangement was not significantly associated with the odds of reporting chronic pain.

### Differences in pain intensity between different disease groups

Pain intensity was most commonly rated as moderate in the four disease groups, with 50.6% of the arthritis group and 39.3% of the COPD group reporting moderate pain in the last 4 weeks. Furthermore, 19.4% of the arthritis group and 15.8% for COPD reported severe pain, while very severe pain was reported by < 2% for all groups. In contrast, the most common pain intensity rating in the non-disease group was no pain (42.0%) (Table 1).

Table 3 shows the differences in pain intensity between different disease groups analyzed by linear regression adjusted for confounding variables such as age, sex, occupational status, living arrangement, lifestyle behavior, and anxiety and depression. People with COPD reported significantly lower pain intensity than people with arthritis and significantly higher pain intensity than people with diabetes or no disease. Pain intensity did not differ significantly between people with COPD and those with heart disease.

**Table 3:** Adjusted linear regression of pain intensity (*n* = 33,955)

*Independent Variables*	Coefficient	SE	*P*-value
*Disease group*			
*Arthritis*	0.45	0.43	< 0.001
*Heart diseases*	- 0.05	0.05	0.356
*Diabetes*	- 0.35	0.06	< 0.001
*No disease*	- 0.47	0.06	< 0.001
*COPD*	reference		
*Sex*			
*Men*	- 0.09	0.02	< 0.001
*Women*	reference		
*Age, years*	0.03	0.004	< 0.001
*Age squared*	- 0.0003	0.00004	< 0.001
*Housing*			
*Living with others*	0.003	0.02	0.877
*Living alone*	reference		
*Occupation*			
*With low educational requirements*	0.09	0.02	< 0.001
*With high educational requirements*	reference		
*Smoking habits*			
*Smoke daily*	0.17	0.02	< 0.001
*Previous smoker*	0.22	0.02	< 0.001
*Smoke occasionally*	0.09	0.03	0.002
*Never smoke*	reference		
*Exercise*			
*Less than once a week*	- 0.15	0.04	< 0.001
*Once a week*	- 0.26	0.04	< 0.001
*2–3 times a week*	- 0.31	0.03	< 0.001
*Nearly every day*	- 0.30	0.04	< 0.001
*Never*	reference		
*Comorbidity*	0.29	0.04	< 0.001
*Hospital Anxiety and Depression Scale (HADS)*			
*HADS anxiety score*	0.06	0.003	< 0.001
*HADS depression score*	0.04	0.004	< 0.001
*Constant*	1.94	0.13	< 0.001

Note: Regression coefficients are unstandardized and the significance is set at *p* ≤ 0.05

Pain intensity was also associated with several other independent variables. Women reported greater pain intensity than men did, and pain intensity increased with increasing age. People employed in occupations with low educational requirements reported greater pain intensity than did those employed in occupations with high educational requirements. Moreover, smoking was associated with greater pain intensity, and exercising was associated with lower pain intensity. People with comorbidity reported higher pain intensity than those without comorbidity, and pain intensity also increased with increasing anxiety and depression scores. Living arrangement was not significantly related to pain intensity.

## Discussion

The findings of this study show a prevalence rate of chronic pain in people with COPD of 51.8%. Those with COPD had higher probability of chronic pain and reported greater pain intensity than both the diabetes and no disease groups, and did not differ significantly from those with heart disease. The arthritis group had significantly higher chronic pain prevalence and pain intensity compared to all other groups. Chronic pain and increased pain intensity were also related to female sex, older age, higher anxiety and depression scores, comorbidity, occupations with low educational requirements, and smoking. On the other hand, exercise seemed to have a protective effect on pain, although these correlational findings could also reflect that pain can be a barrier to physical activity.

### Chronic pain in people with COPD and different medical conditions

The finding that people with arthritis had the highest probability of chronic pain is to be expected because pain is the primary symptom of arthritis [31]. A large European survey on pain that included in-depth interviews with 4839 respondents with chronic pain showed that arthritis was the most common cause of pain (42%) [17]. COPD and heart disease rated next, which generally correspond to findings in a large US cohort, where Roberts et al. found that, after arthritis, patients with COPD were the most likely group to report chronic pain [14]. While we found no significant differences between people with COPD and heart disease, Roberts et al. reported higher rates of chronic pain in COPD than heart disease. While Roberts et al. used specific International Classification of Disease (9th edition) Clinical Modification codes to define COPD and pain, but Centers for Medicare and Medicaid definitions were used to define heart disease, we included a wide range of self-reported heart conditions in the heart disease group. This methodological difference may explain the slight discrepancy between our results and the findings of Roberts et al.

The self-reported comorbidity rate in COPD of 80% was lower than the other disease groups, where heart disease and diabetes had 100% comorbidity. The literature concerning the role of comorbidities in the presence of pain in COPD is conflicting [6]. Nonetheless, COPD is a complex disease that has profound effects on cardiac function and gas exchange with systemic consequences [32]. According to Barnes and Celli (2009), systemic inflammation may also initiate or worsen common comorbid diseases in COPD, such as ischemic heart disease, heart failure, osteoporosis, lung cancer, depression and diabetes [32]. The finding in the present study suggests that comorbidity is highly related to the probability of chronic pain and higher pain intensity in all the non-communicable diseases included in this study.

### Sex differences in reporting of pain

Chronic pain was more common in women than in men, and women also reported greater pain intensity. These findings are consistent with the research literature, which generally reports a higher prevalence of pain in women than in men [33–36]. Other clinical and epidemiological studies have shown that women also have a higher prevalence of many painful disorders, such as rheumatoid arthritis, compared to men [34, 37, 38]. In a study of people with COPD, Christiansen et al. also reported a higher frequency of pain in women than in men [39]. Many explanations for the differences in pain between women and men have been proposed and are supported by evidence; these include neuroimmunological [40], hormonal, genetic, and psychological factors [41]. Moreover, it has been questioned whether men and women construct different meanings of pain, which might influence their reports of the pain experience [42].

### Anxiety and depression

We found higher probabilities of chronic pain and higher pain intensity with increasing anxiety and depression scores. These findings are consistent with prior research showing that higher levels of anxiety are associated with increased clinical pain and heightened experimental pain severity [34]. There is some evidence that the affective dimensions of dyspnea, the main symptom in COPD, such as distress and unpleasantness, are processed in the same areas of the brain that process fear, anxiety, and pain [43, 44], which suggests a possible pathway to explain this association. Our findings suggest that reducing anxiety might be beneficial by also helping patients to reduce their experience of pain.

### Pain and socioeconomic and lifestyle factors

The regression analyses in this study indicate that lower socioeconomic status, which was assessed by the educational requirement for one’s occupation, increased the odds of reporting chronic pain and was associated with greater pain intensity. The research literature shows that the prevalence and intensity of pain are higher among people living in areas of low socioeconomic status [45, 46]. Furthermore, in a study on comorbidity that included 1.75 million patients treated at 341 health centers in Scotland, Barnett et al. [47] found that people living in deprived areas were more likely to have COPD, depression, and pain as comorbidities than other disorders. In that study, among people with COPD, 31% of those living in deprived areas reported having a painful condition, whereas this percentage was reduced by half (15%) for those living in more prosperous areas [47]. In the present study, we had no socioeconomic information other than educational level, but our findings support the relationships between socioeconomic factors and diseases and pain.

The percentage of smokers was high in all disease groups, particularly in those with COPD, in which 76% smoked daily or occasionally. Tobacco smoking has long been acknowledged as the primary cause of COPD in industrial countries [48], and smoking is a recognized risk factor for chronic pain [49]. There is also epidemiological evidence that smokers have higher rates of chronic pain and that they rate their pain as more intense than do nonsmokers [50]. International evidence shows that smoking is more common in groups with low socioeconomic status [51]. Moreover, the bi-directional relationships between smoking and depression in the general population are well established (e.g. [52]). The mechanisms responsible for the relationships between smoking, pain, and depression are complex [50]. In addition to its stimulating effect, nicotine may also have antidepressant effects that maintain smoking [53]. Consequently, the high prevalence of smokers in the present study may suggest that, in people with COPD and other chronic conditions, cigarette smoking represents one way of coping with pain and depression. Many smokers report that smoking improves their mood, suggesting that it may function as self-medication of their depression. The associations between smoking and pain underline the continuing need for tobacco-control policies, which should consider lifestyle and socioeconomic position. Since indoor smoking in public was prohibited in Norway in 2004, smoking habits have changed radically, and the overall percentage of daily smokers has halved [54].

The finding that exercising was inversely related to chronic pain and pain intensity support the available evidence suggesting that physical activity and exercise may improve pain severity [55]. On the other hand, people with pain may also lack the energy to exercise or may find exercise can exacerbate their pain.

### Strengths and weaknesses of the study

The primary strength of the study is the large and unselected sample, which is representative of Norway’s general population. This differs from most previous studies on pain in people with COPD, which have been conducted in clinical, outpatient settings, or pulmonary rehabilitation settings. The participants with COPD may have had other diseases, but we had no information to determine whether COPD was their primary diagnosis. However, by including a variable measuring the presence of comorbidity, we controlled for comorbidity in the regression analyses. We acknowledge some limitations of this study. First, the medical diagnoses relied on self-report, and some morbidities were probably underreported [3]. In the general population, poor agreement between self-report and health administrative data has been reported, particularly in people with COPD [56]. Although the non-disease group probably included individuals with unreported medical conditions, the high number of participants in this group contributed to the high statistical power, which should have neutralized the impact of false negatives. Second, we used employment in a job requiring different levels of education as a proxy for socioeconomic status. Having more details data about educational level and income would have strengthened the validity of our findings. Despite these shortcomings, we believe that the epidemiological data included here will help to corroborate and extend the findings of previous studies on the relationships between diseases including COPD and the experience of pain.

## Conclusion

People with COPD have a high probability of chronic pain and increased pain intensity, second only to those with arthritis among the disease groups included in this study. Multimorbidity, anxiety, and depression, with co-occurring pain, represent a challenge to the health care system. Future pain research should consider assessing for anxiety and depression to further elucidate the effects of these comorbidities on the experience of pain. The relationships between pain and socioeconomic and lifestyle factors, such as occupational level, smoking and exercise, call for continuous efforts in future research and intervention at the societal level to reduce inequality in health.

## Abbreviations

CI: Confidence interval
COPD: Chronic obstructive pulmonary disease
HADS: The hospital anxiety and depression scale
HUNT: Nord-Trøndelag health study
OR: Odds ratio
